# Synthesis and characterization of hyperbranched poly(ester-amine) by Michael addition polymerization

**DOI:** 10.1080/15685551.2017.1351728

**Published:** 2017-07-13

**Authors:** Miao Sun, Chunxiao Yin, Yanan Gu, Yun Li, Zhirong Xin

**Affiliations:** ^a^ School of Chemistry and Chemical Engineering, Yantai University, Yantai, PR China

**Keywords:** Fluorescence, hyperbranched polymers, Michael addition polymerization, tertiary amine

## Abstract

A series of tertiary amine-based hyperbranched poly(amine-ester)s have been synthesized by Michael addition polymerization of trifunctional monomer, TMEA and difunctional monomer, diacylates in chloroform, and the resultant polymers were subsequently treated with mercaptoethenol or 1-dodecanethiol for improving stability in storage. The caption efficiency of mercaptoethanol is much better than that of 1-dodecanthiol. Kinetic study reveals that the thiol group is consumed faster than the acrylate group when the polymerization with feed molar ratio of diacrylate/TMEA = 2/1 was carried out. At initial polymerization, monomer conversion increases fast, but the molecular weights increase slowly and sharp increase of the molecular weight occurs at the final polymerization. The hyperbranched polymers were well characterized by ^1^H NMR spectra and TD-SEC, and DBs of the polymers obtained are between 0.6 and 0.82, as well as the molar ratios of diacrylate/TMEA in the hyperbranched polymers are between 1.60 and 1.82. The fluorescence efficiency and quantum yields of HypET20, HypHT24 and HypDT24 has the following sequence: HypET20 > HypHT24 > HypDT24.

## Introduction

Aliphatic tertiary amine-based polymers display interesting properties and have widely used as absorbents, catalysts, gene and drug carriers [1]. Among this type of polymers, the hyperbranched polymers and dendrimers with tertiary amines attract particularly interests due to their globular architecture and unique properties, such as low toxicity, pH-sensitivity, fluorescence and abundant surface functional groups [2]. Poly(ester amine)s, which are prepared by Michael addition polymerization of diacrylate and trifunctional amines, are biodegradable, and have been widely used in gene delivery with high transfection efficiency, importantly, the results reveal no or low cytotoxicity [3]. The hyperbranched poly(amido amine) (HPAMAM) prepared from trifunctional amines and diacrylamide have the same application, and also can be used as drug carrier [4]. Although molecular structure of the HPAMAM is irregular in comparison with the dendrimers, they demonstrate unique characteristics or advantages in supra molecular self-assembly behaviors [5]. As a widely used polycationic nonviral gene vector, the polyethylenimines obtained after appropriate surface modification reveal high transfection efficiency and low toxicity [6]. Recently, these hyperbranched polymers and dendrimers were found to emit fluorescence under irradiation of approximately 375 nm [7]. Since they do not have traditional chromophore, where the fluorescence comes from becomes a problem. We proposed that the fundamental chromophore is the tertiary amine in the branched units based on quenching investigation of the functional groups in the HPAMAM [8]. The previous fluorescence-quenching study of the PAMAM dendrimers revealed that the fluorescence originates from the interior region, and the fluorescence of surface area was not observed [9], and these two results of study are not contrary because the tertiary amine groups in the branching units are located in the interior region of the hyperbranched molecules. Imae assumed that the oxygen-doped tertiary amine is chromophore because oxidation of PAMAM dendrimers can increase the fluorescence [10]. To clarify what is the fundamental chromophore in these hyperbranched polymers or dendrimers, it is better to synthesize the polymers directly from the tertiary amine monomers, resulting in the polymers only with the tertiary amine because the primary and secondary amines cannot emit fluorescence due to predissociation [11]. However, all the hyperbranched polymers and dendrimers presented above generally contain primary and secondary amines besides tertiary amine because they are prepared by Michael addition polymerization of multifunctional acrylates, or acrylamide and primary or/and secondary amines [12,13], and the tertiary amines are formed during the reactions, incomplete reaction will produce the primary and secondary amines. To ensure only tertiary amine groups in the hyperbranched polymers, the best synthetic strategy is synthesis of the polymers directly from tertiary amine monomers. Unfortunately, such synthetic strategy has not been reported except our recently published report [14] based on our knowledge.

Michael addition polymerization is a versatile synthetic methodology of polymers ranging from linear to hyperbranched polymers [15]. Instead of amine, thiol can also be used as heteroatomic donor in Michael addition reaction [15], and the reaction of SH with acrylate has been used to prepare the polymers [16]. In this article, we study the synthesis of hyperbranched poly(ester-amine)s with only tertiary amine just by linking tertiary amines together via Michael addition reaction of thiol and acrylate.

## Experimental part

### Materials

Triethanolamine, thionyl chloride, thiourea, chloroform (analytical grade) and mercaptoethanol containing ~10 mol% bis(2-hydroxyethyl) were purchased from Sinopharm Chemical Reagent Co. Ltd. and used as received. Acryloyl chloride (≥96%, Aladdin Reagent) was purified by distillation under reduced pressure before use. Ethylene glycol diacrylate (EGDA) was prepared according to our previous method [14]. ^1^H NMR (CDCl_3_, pp): 6.40, 6.15 (4H, C*H*H=C*H*CO–); 5.87 (2H, CH*H*=CHCO–); 4.40 (4H, –OC*H*_2_C*H*_2_O–).

### Characterization

#### Nuclear magnetic resonance (NMR)

Structures of the synthesized compounds were characterized by ^1^H and ^13^C NMR spectra on a Bruker 400 MHz NMR spectrometer using CDCl_3_, DMSO-*d*_*6*_ or D_2_O as solvent.

#### Size exclusion chromatography (SEC) measurements

Molecular weight and molecular weight distribution were obtained by triple detection size exclusion chromatography (TD-SEC) detection at 25 °C_._ The instrumentation consists of the following: a Waters 1515 isotratic HPLC pump with 5 μm Waters styragel columns (guard, 0.5 HR, 1 HR, 3 HR, 4 HR and 5 HR, the molecular weight ranges of these columns are 0–1000, 100–5000, 500–30,000, 5000–500,000 and 50,000–4,000,000 g/mol, respectively); a Waters 717 PLUS autosamples; a waters 2414 differential refractive index (DRI) detector, the wavelength is 880 nm; a multi angle laser light scattering (MALLS) detector (Wyatt mini Dawn TRISTRA light scattering, three detection angles are 45°, 90° and 135°, the wavelength and power are 690 nm and 220 w); a Wyatt Visco Star viscometer detector; a Waters Breeze data manager. The eluent was HPLC grade THF delivered at 1.0 mL/min. The refractive index increment (dn/dc) was determined using a Wyatt Optilab REX (*λ* = 640 nm) interferometric differential refractometer in bath model at 25 °C.

#### Measurement of fluorescence spectra

The fluorescence spectra of the resultant polymers were recorded at room temperature on a Perkin-Elmer LS55 luminescence spectrophotometer under excitation wavelength of 375 nm, in CHCl_3_.

#### The quantum yield

The quantum yield of the polymers was calculated according to the following equation:

$$\Phi \text{SA}=\Phi \text{ST}\left(\text{SSA}/\text{SST}\right)(\eta \text{SA/}\eta {\text{ST)}}^{\text{2}}$$

where Φ = quantum yield; S = gradient of the curve obtained from the plot of intensity vs. absorbance; *η* = refractive index of the solvent; SA = the sample, and ST = the standard. Anthracene (quantum yield = 0.305 in CHCl_3_) was used as a standard. The polymers and anthracene were all dispersed in CHCl_3_. The slit width kept the same for both the standard and the samples. Absorbance was measured on a Shimadzu UV-2401PC spectrophotometer.

#### Synthesis of tris(2-mercaptoethyl)amine (TMEA)

Synthesis of TMEA includes three steps. The first step is synthesis of tris(2-chloroethyl)amine. A solution of triethanolamine (29.8 g, 0.2 mol) in 50 mL of chloroform was dropwise added into a solution of thionyl chloride (52 mL, 0.7 mol) in chloroform (80 mL) in a 250 mL two-necked round-bottom flask, which was fitted with a dropping funnel and a reflux condenser, while stirring. The addition was carried out at ambient temperature for 1 h, the reaction continued at room temperature until gas evolution stopped, and then the mixture was heated to refluxed temperature for 4 h. After cooling to room temperature, the white solid product was filtered and washed with 3 × 50 mL of chloroform, and then the tris(2-chloroethyl)amine hydrochloride was obtained in 80% yield after dried in a vacuum oven at room temperature for 24 h.

The second step is the preparation of tris(ethylisothiouronium)amine chloride. A mixture of tris(2-chloroethyl)amine hydrochloride (36.15 g, 0.15 mol) and thiourea (34.3 g, 0.45 mol) in 100 mL of ethanol was refluxed at 80 °C for 5 h. After cooled to room temperature, the tris(ethylisothiouronium)amine chloride was obtained in 90% yield by filtration and following vacuum drying.

The third step is synthesis of TMEA. A solution of tris(ethylisothiouronium)amine chloride (37.3 g, 0.08 mol) in 80 mL of water was bubbled with N_2_ for 10 min. After sodium hydroxide (20 g, 0.5 mol) was added, the reaction mixture was heated at 100 °C for 3–5 min under N_2_ atmosphere. The solution was quickly cooled to 20 °C in an ice bath, and then neutralized by slowly adding the diluted hydrochloric acid solution. The solution was extracted with 3 × 25 mL of chloroform. The extracts were dried over anhydrous magnesium sulfate, after filtration, the solvent was removed in *vacuo.* The pure product TMEA was obtained in 45% yield as colorless oil by subsequent distillation. ^1^H NMR (CDCl_3_, ppm, TMS): 2.83–2.50 (12H, HSC*H*_2_C*H*_2_–); 1.75 (3H, *H*SCH_2_CH_2_–); ^13^C NMR (CDCl_3_, ppm): 57.08 (CH_2_ *C*H_2_–); 23.04 (HS*C*H_2_–); HRMS (EI+): m/z calcd for C_6_H_15_NS_3_: 197.388, found: 198.0442.

#### Synthesis of hexamethylene glycol diacrylate (HGDA)

The synthesis procedure is similar to that of EGDA. Hexamethylene glycol (11.8 g, 0.1 mol) and triethylamine (TEA, 25.3 g, 0.25 mol) in 80 mL of anhydrous dichloromethane were added to a 250 mL round-bottom flask equipped with a magnetic stirrer. Acryloyl chloride (22.6 g, 0.25 mol) dissolved in 20 mL anhydrous dichloromethane were introduced dropwise to the stirred solution for 45 min under N_2_ at 0 °C. Then the solution was warmed to room temperature and was stirred overnight. After the salt of TEA was filtered, the filtrate was washed with aqueous solutions of sodium bicarbonate and sodium chloride three times, respectively. The organic layer then was dried over anhydrous magnesium sulfate and filtered. After removing the solvent in *vacuo*, the oil product was purified by silica chromatography using dichloromethane/petroleum ether solution as eluent. Finally, HGDA was obtained in 35% yield. ^1^H NMR (CDCl_3_, ppm, TMS): 6.36 and 6.12 (4H, C*H*H=C*H*CO–); 5.79 (2H, CH*H*=CHCO–); 4.17 (4H, –OC*H*_2_CH_2_CH_2_–); 1.68 (4H, –OCH_2_C*H*_2_CH_2_–); 1.42 (4H, –OCH_2_CH_2_C*H*_2_–).

#### Synthesis of decamethylene glycol diacrylate

The synthesis procedure was similar to that of HGDA. Decamethylene glycol (17.4 g, 0.1 mol) and triethylamine (TEA, 25.3 g, 0.25 mol) in 80 mL of anhydrous dichloromethane were added to a 250 mL round-bottom flask equipped with a magnetic stirrer. Acryloyl chloride (22.6 g, 0.25 mol) dissolved in 20 mL anhydrous dichloromethane were introduced dropwise to the stirred solution for 45 min under N_2_ atmosphere at 0 °C. Then the solution was warmed to room temperature and was stirred overnight. After the salt of TEA was filtered, the filtrate solution was washed with sodium bicarbonate and sodium chloride solution three times. The organic layer was dried over anhydrous magnesium sulfate and filtered. After removing the solvent in vacuo, the oil product was purified by silica chromatography using dichloromethane/petroleum ether solution as eluent, and final product was obtained in 38% yield. ^1^H NMR (CDCl_3_, ppm, TMS): 6.42 and 6.09 (4H, C*H*H=C*H*CO–); 5.82 (2H, CH*H*=CHCO–); 4.15 (4H, –OC*H*_2_CH_2_CH_2_CH_2_ CH_2_–); 1.67 (4H, –OCH_2_C*H*_2_CH_2_CH_2_ CH_2_–), 1.25–1.43 (8H, –OCH_2_CH_2_C*H*_2_C*H*_2_ CH_2_–).

#### Synthesis of hyperbranched poly(amine-ester)s (HypETs)

TMEA (0.29 g, 1.5 mmol), EGDA (0.51 g, 3 mmol) and 1.5 mL chloroform were successively added into a 5 mL glass tube with a magnetic bar, and then the system was degassed by three freeze-pump-thaw cycles. The tube was sealed under vacuum, and then the sealed tube was placed in an oil bath at 50 °C. After the polymerization was carried out for 15 h, or 20 h or 24 h, the tube was cooled to room temperature and opened. Into the reaction mixture, the mercaptoethanol (1.13 g) or 1-dodecanethiol (1.518 g) was added, and the tube was sealed again after three freeze-pump-thaw cycles. The reaction was carried out for additional 24 h at 50 °C. After cooling to room temperature, the tube was opened, and the solution was poured into diethyl ether while stirring vigorously, the polymer was precipitated. After filtration and drying in vacuo at room temperature for 24 h, the target hyperbranched polymers, which were respectively marked as, HypET_ME_15, HypET_ME_20 and HypET_ME_24 for the polymers obtained from polymerization for 15, 20 and 24 h and then treated with mercaptoethanol, were obtained.

The HypHT24 and HypDT24 were prepared by the same procedure as the preparation of HypETs except the recipes, for preparation of HypHT24, TMEA (0.29 g, 1.5 mmol), HGDA (0.68 g, 3 mmol) and 1.5 mL chloroform were used and final product was obtained in 76% yield; for HypDT24, TMEA (0.29 g, 1.5 mmol), DGDA (0.9 g, 3 mmol) and 1.5 mL chloroform were used, and finally, the HypDT24 was obtained in 61% yield.

## Kinetics study of polymerization

TMEA (0.12 g, 0.6 mmol), EGDA (0.20 g, 1.2 mmol), 0.6 mL CDCl_3_ and 0.1 g dichloromethane (as an internal standard) were successively added into a 5 mL glass tube with a magnetic bar, and then the system was degassed by three freeze-pump-thaw cycles. The tube was sealed under vacuum, the sealed tube was placed in an oil bath at 50 °C. After a predetermined interval, the NMR spectra were measured. The conversions of thiol and acrylate were calculated based on the NMR data.

## Results and discussion

**Scheme 1.**
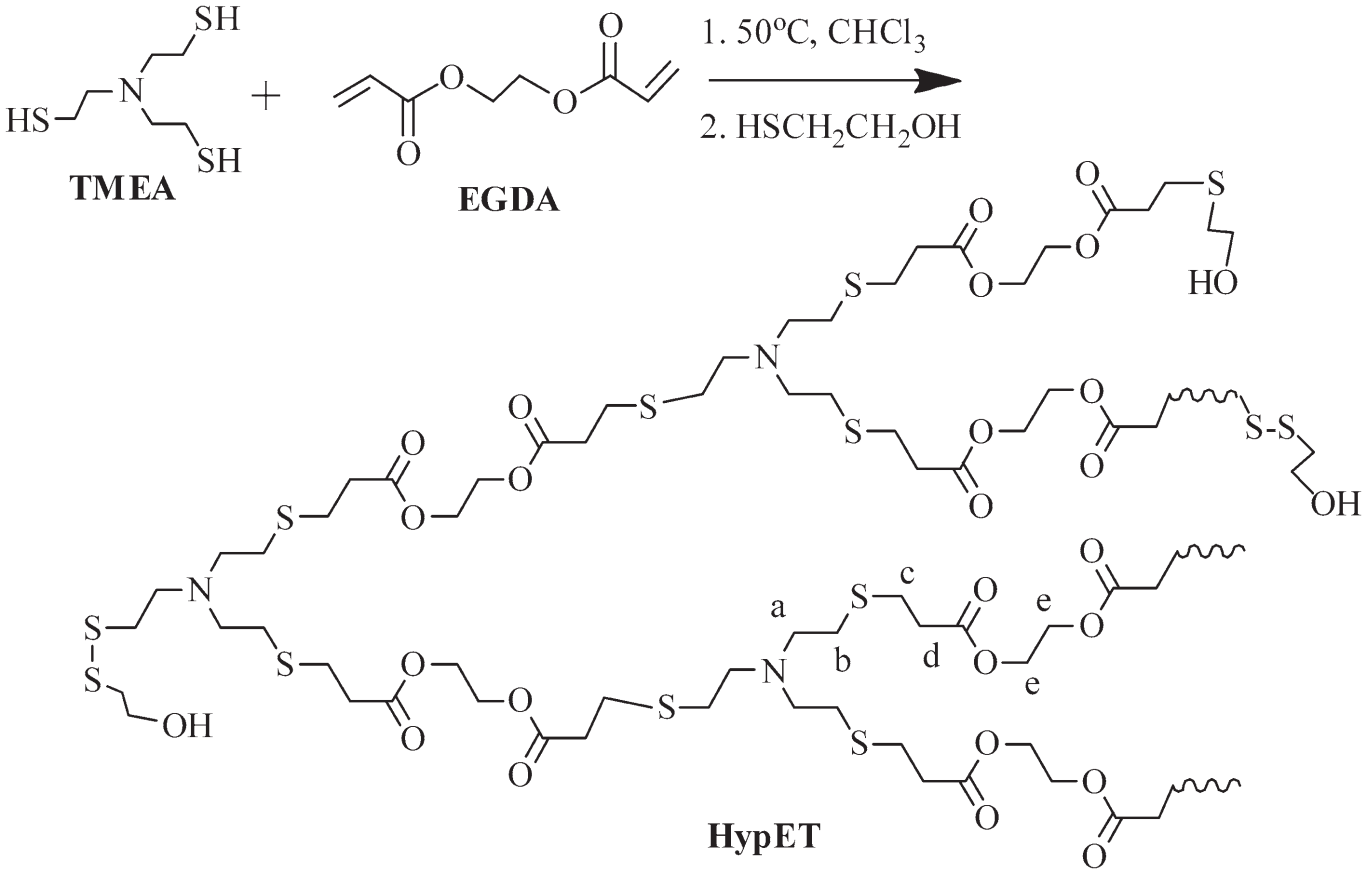
Preparation of HypET by Michael addition polymerization of TMEA and EGDA.

Hyperbranched polymers are typically prepared using AB2 monomer [17]. Since the reactivity of the reaction between thiol and acrylate is very high, synthesis of the tertiary amine AB_2_ monomers with single (or two) acrylate group and two (or single) thiol groups is very difficult. Therefore, an A3 + B2 polymerization system, TMEA with three terminal thiol groups and EGDA with two acrylate groups, was used as monomers in this study, and the polymerization proceeds according to Scheme 1.

**Scheme 2.**
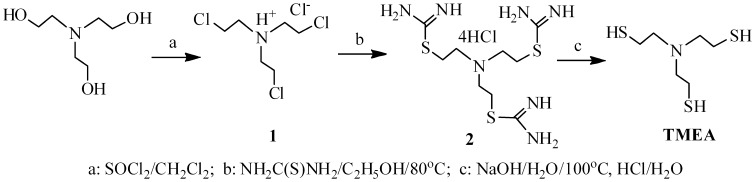
Preparation of tris(2-mercaptoethyl) amine.

In the synthesis of tertiary amine-based hyperbranched polymers, a key monomer is TMEA, which was prepared from triethanolamine by transformation of the terminal hydroxyl to thiol group via three steps according to Scheme 2, but three reactions are fulfilled in one-pot, separation of the intermediates 1 and 2 is not needed. The resultant product was well characterized by ^1^H and ^13^C NMR spectra, and mass spectrum as presented in the experimental section.

### Michael addition polymerization of TMEA and EGDA

It is one problem to solve how to avoid the crosslinking reaction during the polymerization for A_2_ + B_3_ system. Based on the Statistical approach for estimation of gel point [18], the molar ratio of EGDA/TMEA should exceed 1.91 when the extent of reaction equals 0.90. When the polymerization with feed molar ratio of EGDA/TMEA = 1.51 or 1 was carried out at 50 °C in chloroform, the gelation occurred after less than 22 h or 17 h polymerization (HypETg1 or HypETg2 in Table 1). However, for polymerization with molar ratio of EGDA/TMEA = 2.0, no gelation was taken place even after 24 h polymerization (Table 1). Thus this molar ratio of EGDA/TMEA was used in the kinetics study.

**Table 1.** Polymerization conditions and results for Michael addition polymerization of diacrylate esters and TMEA.

No^a^	Time (h)	DA/TMEA (molar ratio)	Yield^b^ (%)	*M*_w_^c^ (g/mol)	*M*_w_/*M*_n_^c^	DB^d^	DA/TMEA^e^ (molar ratio)	Reaction efficiency^f^
HypET_ME_15	15	2/1	63	14,800	2.64	0.79	1.82	0.99
HypET_ME_20	20	2/1	66	20,900	3.54	0.80	1.83	0.99
HypET_ME_24	24	2/1	70	55,100	7.06	0.82	1.84	0.99
HypETg1	<22	1.5/1	gel	–	–	–	–	–
HypETg2	<17	1/1	gel	–	–	–	–	–
HypET_DT_13	13	2/1	35	15,690	2.12	0.60	1.62	0.79
HypET_DT_29	29	2/1	45	21,560	2.01	0.79	1.82	0.82
HypHT24	24	2/1	76	43,300	3.69	0.73	1.78	0.99
HypDT24	24	2/1	61	35,900	2.10	0.70	1.75	0.98

^a^ Hyperbranched polymers were prepared by Michael addition polymerization at 50 °C in CHCl_3_ for various times and subsequently treated by mercaptoethanol for HypET_ME_, or treated by 1-dodecanethiol for HypET_DT_; DAs in HypHT and HypDT is hexamethylene glycol diacrylate and decamethylene glycol diacrylate.

^b^ Obtained by weight method.

^c^ Molecular weights and molecular weight distributions were measured by triple detection size exclusion chromatography.

^d^ Calculated based on 1H NMR data.

^e^ Molar ratio of DA/TMEA in the hyperbranched polymers, which was calculated based on ^1^H NMR data.

^f^ Reaction efficiency between thiol and acylate was calculated based on ^1^H NMR data.

**Figure 1.**
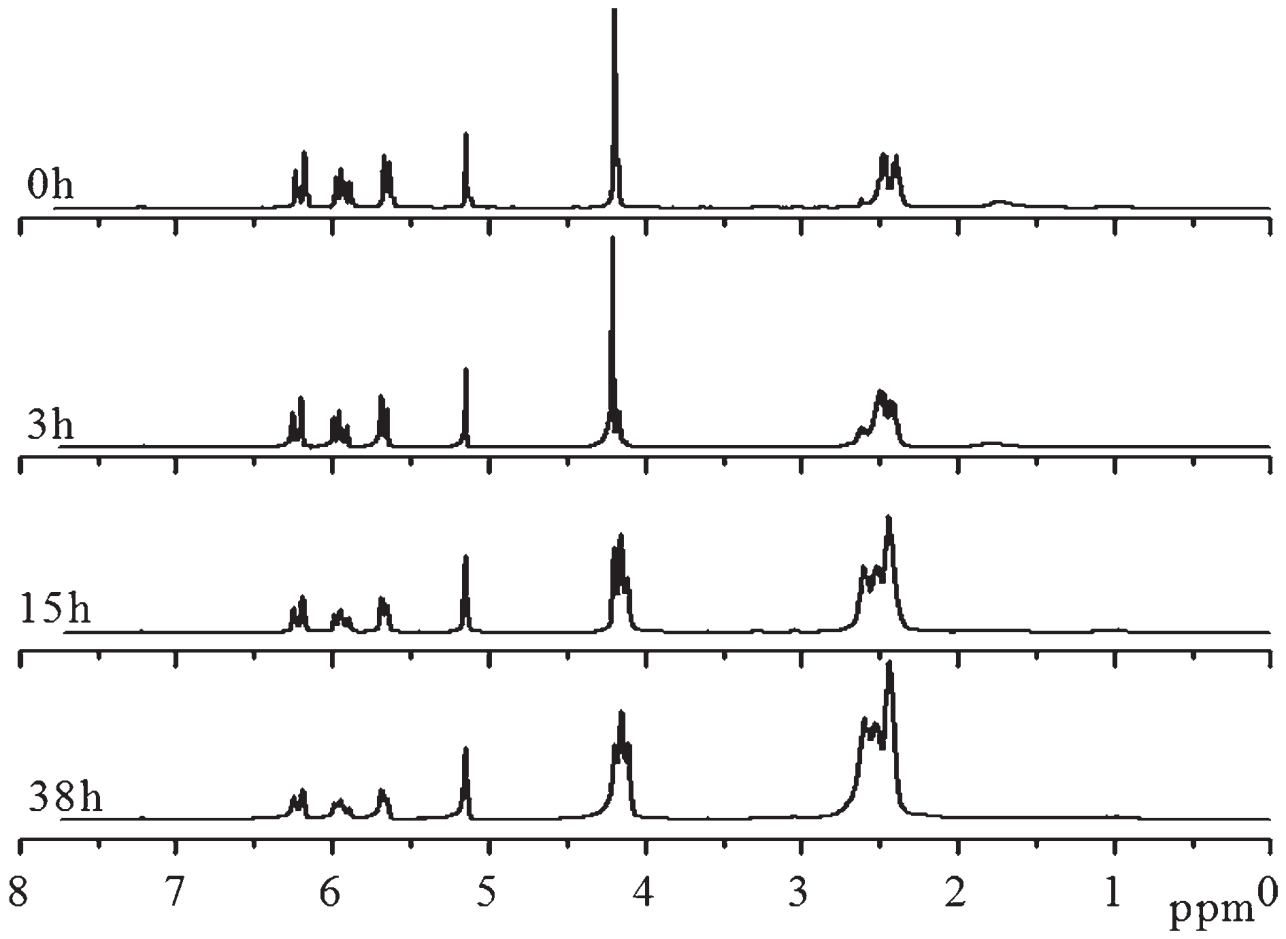
^1^H NMR spectra of the HypETs formed by polymerization with feed molar ratio of EGDA/TMEA = 2/1 at 50 °C in chloroform for 0, 3, 15 and 38 h, respectively.

**Figure 2.**
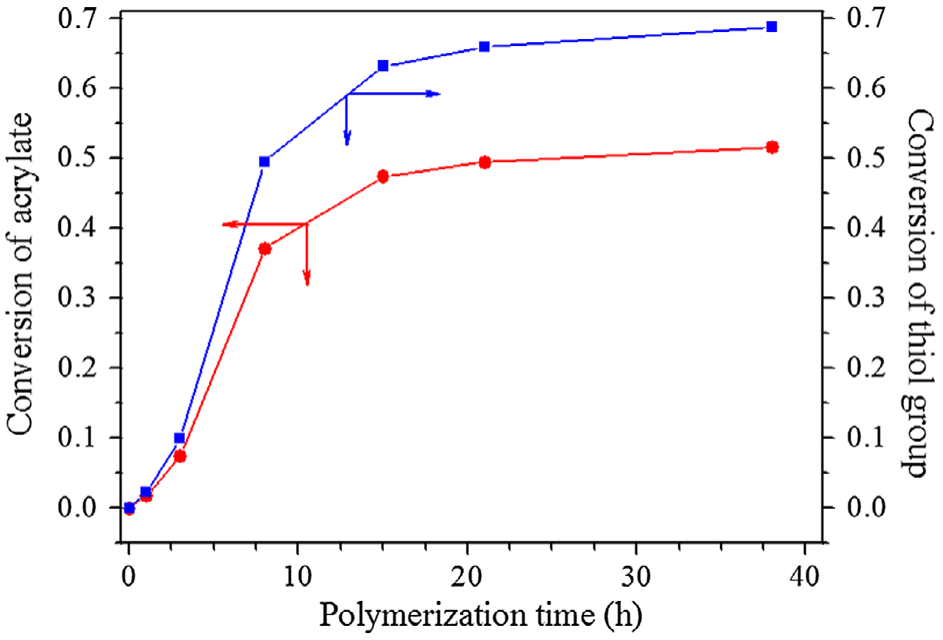
Conversions of thiol and acrylate with polymerization time in the polymerization with EGDA/TMEA = 2/1 at 50 °C in chloroform for different reaction time.

For ensuring the hyperbranched polymerization stopped before the crosslinking reaction, it is necessary to estimate the polymerization time, when conversion of thiol group reached 90% in the polymerization of EGDA and TMEA. Kinetics of the polymerization was studied by ^1^H NMR with polymerization proceeding in NMR tube using CDCl_3_ as solvent, and the four typical NMR spectra are presented in Figure 1. Quantitative dichloromethane was added into the polymerization system as internal standard for calculation of thiol and acrylate conversions, and its proton signal appears at = 5.15 ppm in Figure 1. We can see that the signals at = 5.66, 5.94 and 6.20 ppm ascribed to vinyl protons decrease with increasing polymerization time, and their integral values was used to calculate the conversion of acrylate. The ester methylene proton signal of EGDA at around 4.14 ppm in the polymers is shifted slightly from the EGDA monomer, but the integration ratios of the signal at = 4.22–4.06 to the signal at = 5.15 ppm are not varied in the polymerization. The proton signals at = 2.31–2.71 ppm are strengthened with increasing reaction time as shown in Figure 1, which is the result of reaction between thiol and acrylate, and two methylene protons signals of the newly formed SCH_2_CH_2_COO are shifted from = 5.66, 5.94 and 6.20 ppm to 2.31–2.71 ppm, thus, their integral value increase was used to calculate the conversion of thiol groups, and the results are shown in Figure 2. We can see that the conversions of both thiol and acrylate groups increase fast before 15 h polymerization, and then conversion increase levels off. However, the conversion of thiol did not exceed 80% even though polymerization lasted for 38 h. In addition, the solution polymerization in chloroform is also important to suppress the gelation of polymerization system, and when this polymerization was carried out in bulk the gelation occurred. We emphasize here that the conversion in Figure 2 refers to functional groups conversion, not to monomers conversion because the thiol and acrylate groups detected can be in the polymer chains, also in the monomers. Because in the thiol-ene addition reaction 1 mol –SH group consumed 1 mol acrylate group, conversion of –SH group is always higher than the conversion of acrylate group (Figure 2) for the polymerization system with [CH_2_=CH]/[SH] = 4/3 is easily understood.

**Figure 3.**
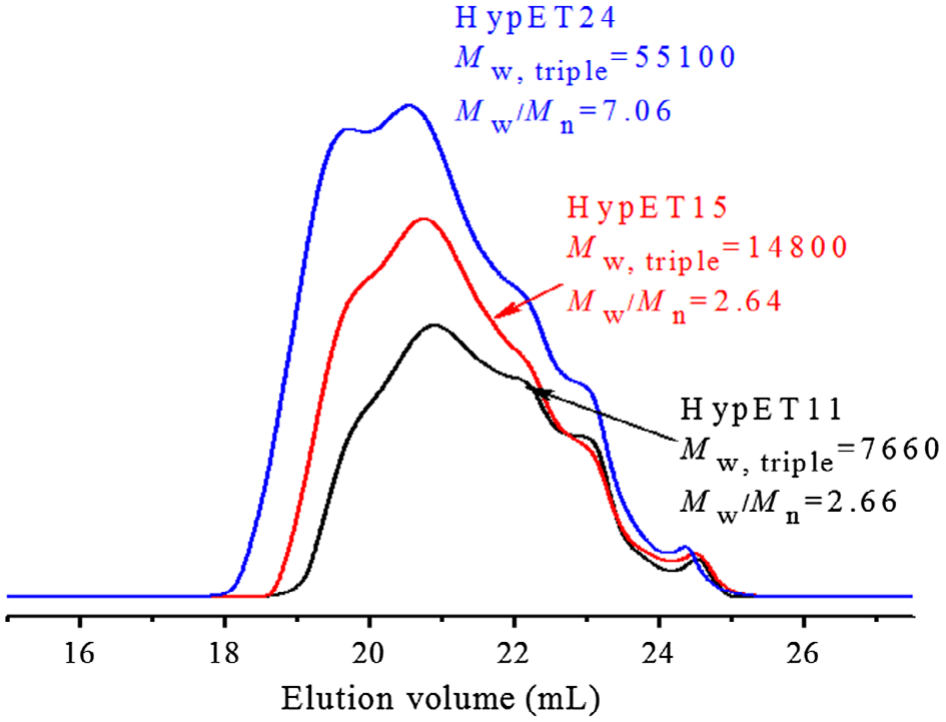
SEC-TD traces of HypET11, HypET15 and HypET24, which were prepared by Michael addition polymerization with molar ratio of EGDA/TMEA = 2/1 in chloroform.

**Figure 4.**
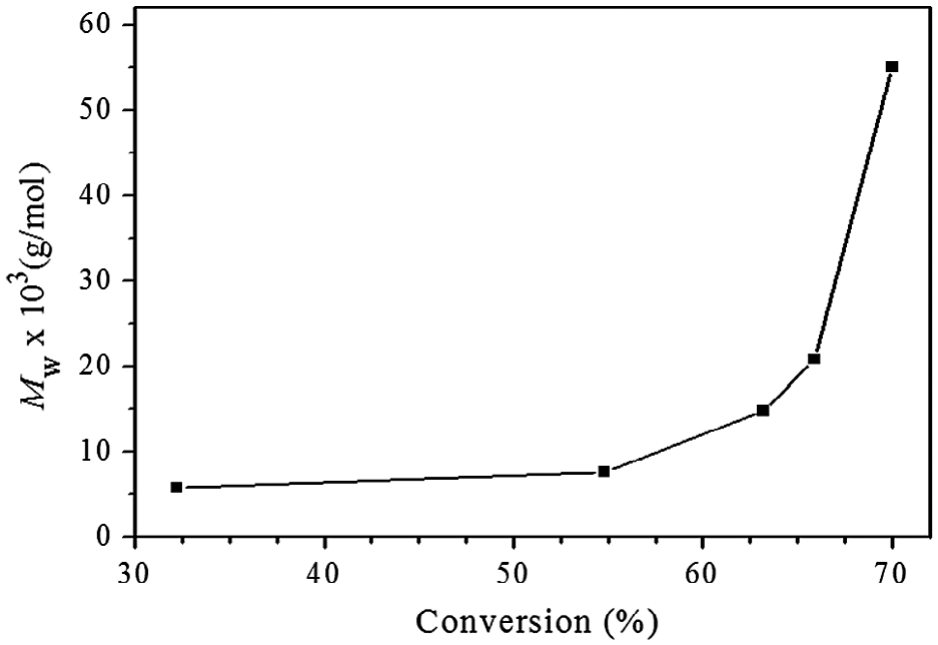
Relationship of *M*_w_, triple with conversion for Michael addition polymerization of EGDA and TMEA with feed molar ratio of EGDA/TMEA = 2/1 in chloroform at 50 °C.

To further understand the polymerization kinetics, SEC-TD was used to follow the polymerization, and three typical SEC curves are presented in Figure 3, the relationship of molecular weight with the conversions is shown in Figure 4. We can see in Figure 4 that the molecular weights of the resultant macromolecules increase slowly at the initial stage of polymerization, significant molecular weight increase occurs at the last stage of polymerization, which is general phenomenon in the polymerization of AB_*f*_ (*f* ≥ 2) monomer. As we know, the initial reactions take place mainly between monomers, and/or among the monomers and low oligomers, resulting in slow increase of the molecular weight at initial stage of polymerization. When the most of monomer molecules are consumed the reactions between macromolecules may occur, which causes sharply increase of the molecular weights at latter polymerization stage. Figure 3 displays an interesting phenomenon, with proceeding of the polymerization, molecular weights of the high molecular weight polymer parts increase fast, while lower molecular weight polymers parts decrease slowly, but do not disappear. This indicates that the high molecular weight polymers are much more reactive than the oligomers due to their numerous surface functional groups. And higher molecular weight macromolecules can react with each other, which leads to sharp increase of molecular weight compared to the oligomers and monomers, as shown in Figure 4.

**Figure 5.**
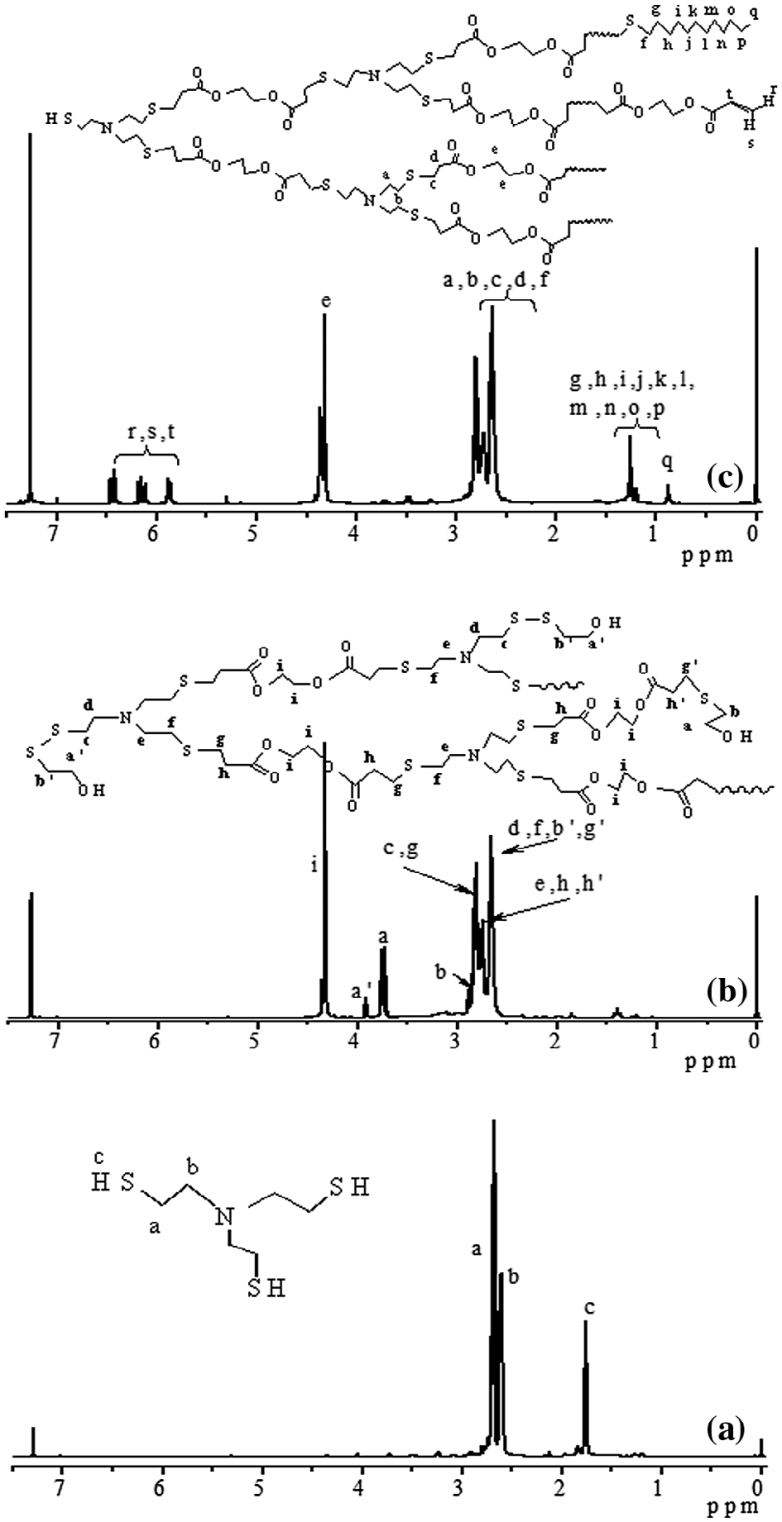
^1^H NMR spectra of tris(2-mercaptoethyl)amine (a), HypETME15 prepared by polymerization with molar ratio of.

**Figure 6.**
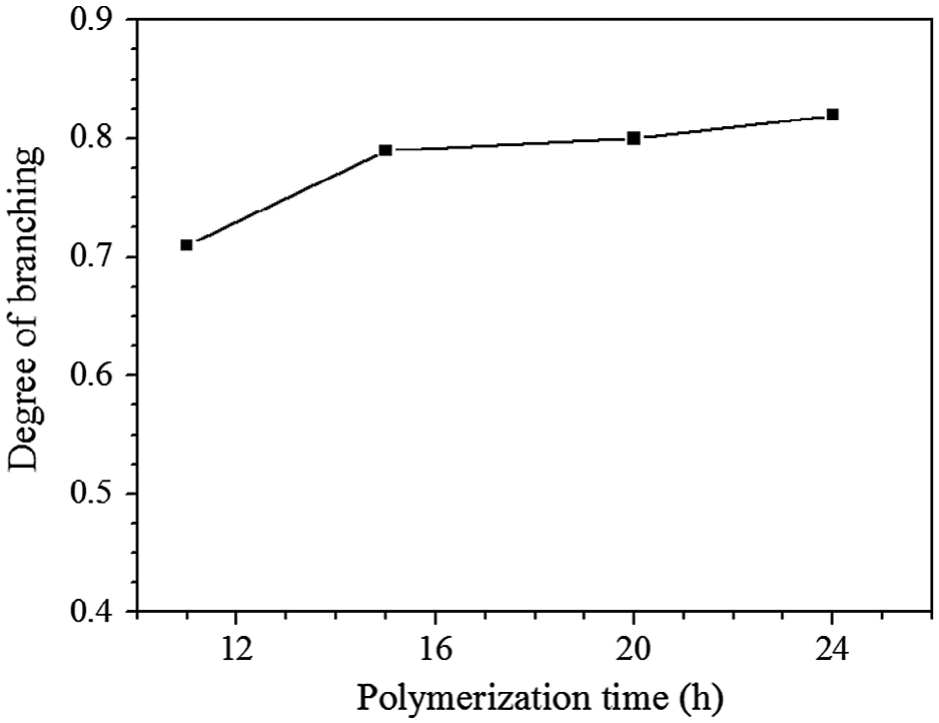
Influence of polymerization time on degree of polymerization for Michael addition polymerization with feed molar ratio of EGDA/TMEA = 2/1 in chloroform at 50 °C.

**Figure 7.**
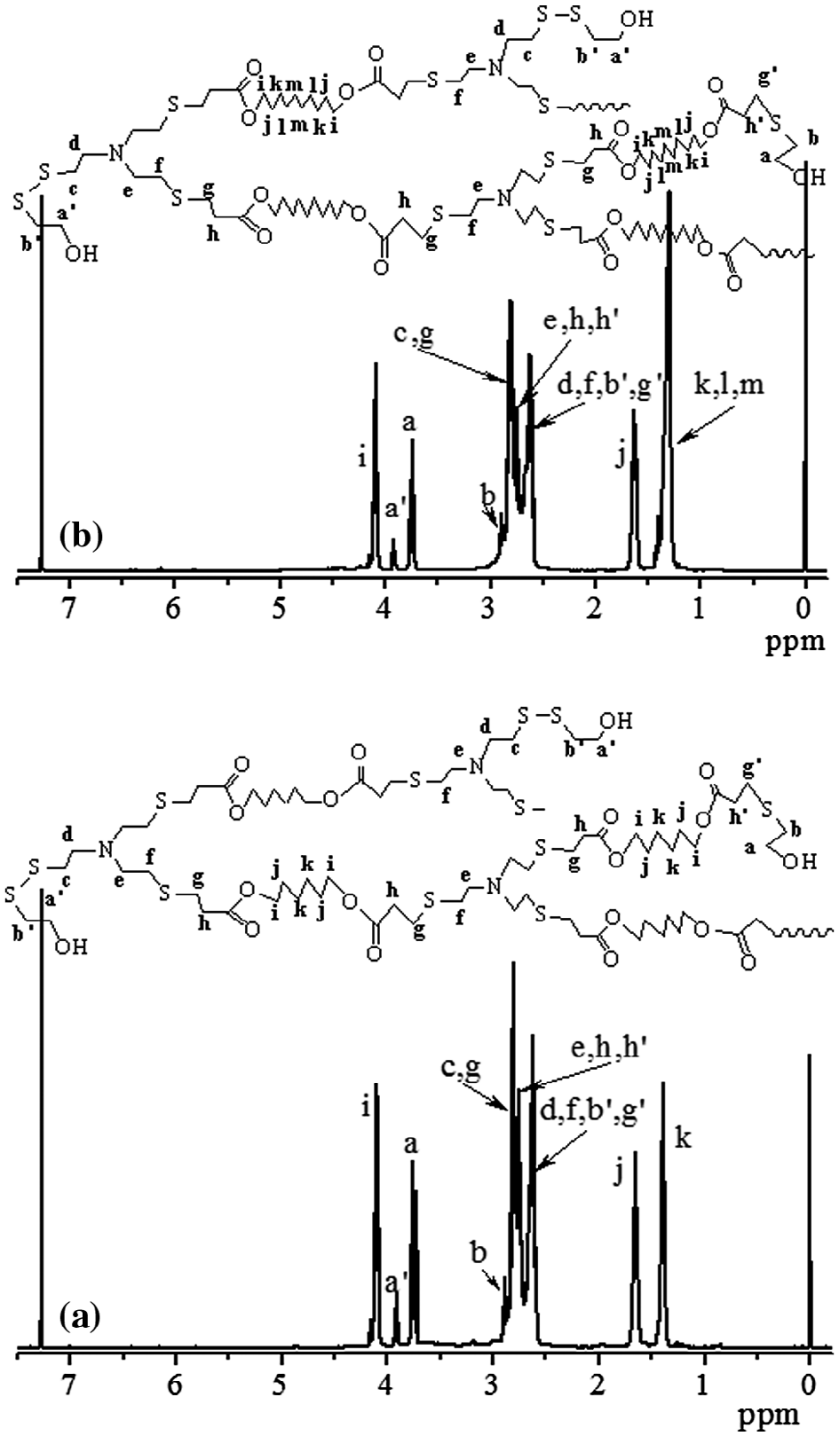
^1^H NMR spectra of HypHT24 (a) and HypDT24 (b), which were prepared by Michael addition polymerization of TMEA.

**Figure 8.**
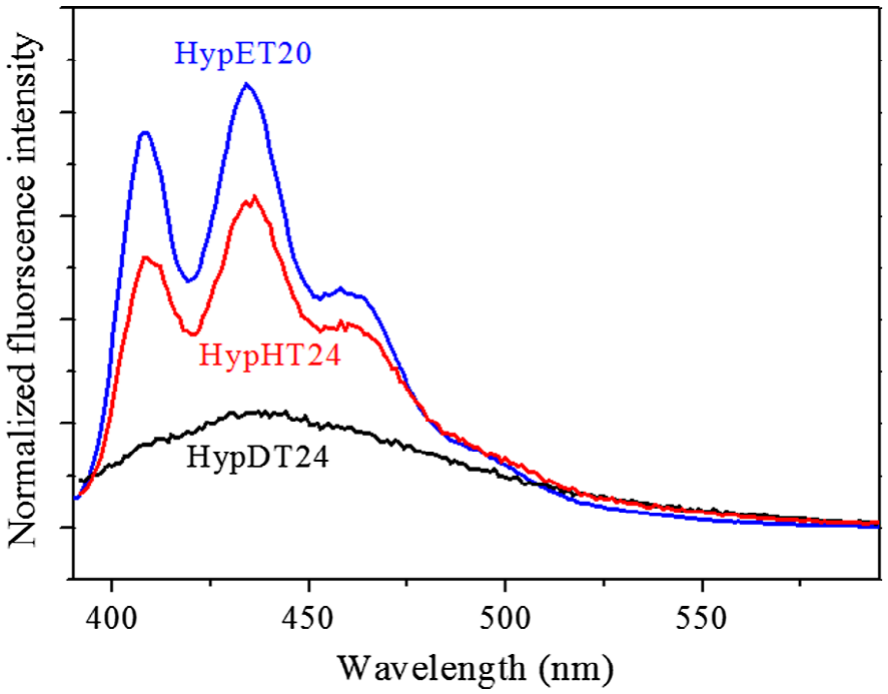
Fluorescence spectra of the HypET20, HypHT24 and HypDT24, which were prepared by Michael addition polymerizations of TMEA respectively with EGDA, HGDA.

**Figure 9.**
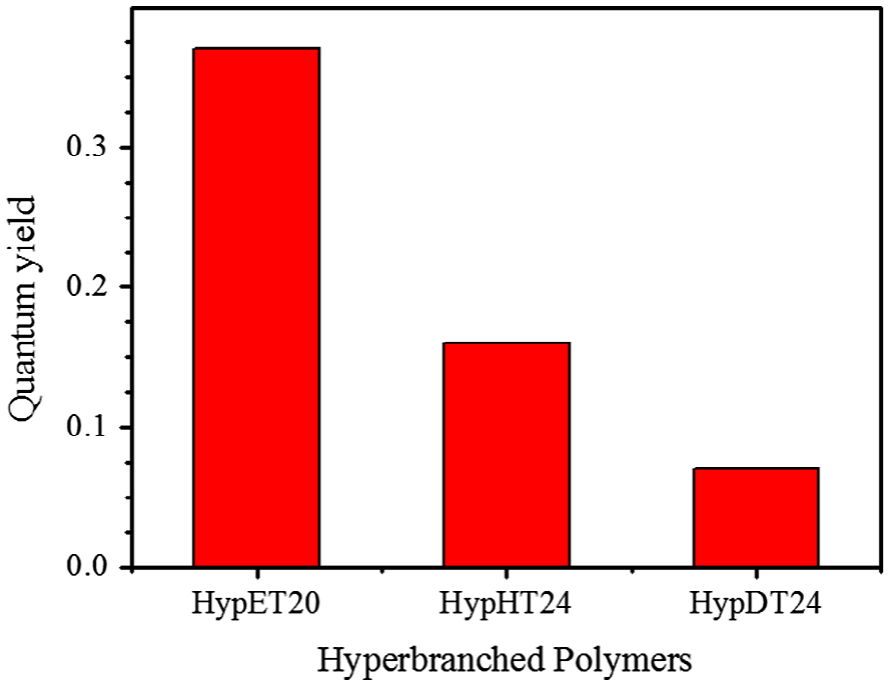
Influence of the hyperbranched structure on the quantum yields.

### Structure analysis of hyperbranched polymers

When the HypETs were prepared by Michael addition polymerization of TMEA and EGDA with molar ratio of 1:2 at 50 °C for different times, the resultant hyperbranched polymers are not stable during the storage, because two kinds of active terminal groups, thiol and acrylate, exist on their surface, and their further reaction can forms crosslinked polymers. Therefore, after the polymerization ended, the polymer solution was further treated with mercaptoethanol or 1-dodecanethiol, and the corresponding hyperbranched polymers are marked as HypET_ME_ and HypET_DT_, respectively. Since the thiol can react with terminal acrylate group via Michael addition reaction and also can react with terminal thiol via oxidation reaction [14,19], both reactions produce the same terminal hydroxyl group (or C_12_H_25_ group) when the polymer solution is treated with mercaptoethanol (or 1-dodecanethiol) as shown in Scheme 1. This is supported by their ^1^H NMR spectra shown in Figure 5. As shown in Figure 5(b), for the HypET treated with mercatoethanol, the vinyl proton signals at = 5.86, 6.15 and 6.44 ppm almost disappear, which demonstrates high efficiency of these two reactions, and two new proton signals appear at = 3.91 (a′) and 3.74 (a) ppm, which are ascribed to two different methylene protons next to terminal hydroxyl group, which are respectively formed by the reaction of terminal SH with mercaptoethanol to produce S–SCH_2_CH_2_OH group; and by the reaction of terminal acrylate with mercaptoethanol to yield CH_2_–SCH_2_CH_2_OH group. The results support our previous presumption that the obtained hyperbranched polymers have two types of terminal groups, thiol and acrylate, and the linear and terminal structures in the HypETs are formed. Based on the integral values of the signals at = 5.86 and 3.74 ppm, the reaction efficiencies between thiol and acrylate were calculated and the results are listed in Table 1, all reaction efficiencies are approximately 99%. However, when the HypET was treated with 1-dodecanethiol, ^1^H NMR spectrum of the resultant product in Figure 5(c) reveals significant decrease of the vinyl proton signals at = 5.87, 6.15 and 6.44 ppm, but these signals can be seen clearly in Figure 5(c). And the signal at = 0.87 ppm is ascribed to methyl protons of the terminal dodecyl, which is formed by reaction of 1-dodecanethiol with the terminal acrylate, the reaction efficiencies were calculated based on the integral values of the signals at = 5.87 and 0.87 ppm, and approximately 80% of the reaction efficiencies (Table 1) reveal that the 1-dodecanethiol is not as reactive as mercaptoethanol during the reactions with acrylate probably due to their different structures.

As we mentioned above, the feed molar ratio of EGDA/TMEA was 2/1, but composition of hyperbranched polymers measured by 1H NMR spectra are different. The proton signals at = 4.33, 2.53–2.94 and 3.65–3.97 ppm are ascribed to ester methylene group of EGDA, two methylene groups next to nitrogen and sulfur as well as methylene group adjacent to terminal hydroxyl group. Based on the integral values of these signals, the molar ratios of EGDA/TMEA units in the HypETs obtained from different polymerization times were calculated, and the results are listed in Table 1. The ratios increase slightly with increasing polymerization time, but all the molar ratios (around 1.80) of EGDA/TMEA units in the hyperbranched polymers are less than 2 of the feed molar ratio. This result implies existence of some thiol groups in the polymers besides one terminal thiol group in every macromolecule, thus, there are linear units in the polymers.

The degree of branching (DB) is an important parameter of the hyperbranched polymers and can be calculated according to equations, DB = (D + T)/(D + T + L) suggested by Fréchet et al. [20], where D, T, and L refer to the contents of dendritic, terminal, and linear units, respectively. Based on the integration of the signals at = 3.90 (a′) and 3.74 ppm (a), we can calculate the DB of the HypETs, and the results are shown in Figure 6. Prolonging reaction time can increase the DB of HypETs formed, but only limited increase of DB is possible based on the result that the DB increases slightly with increasing polymerization time shown in Figure 6.

Synthesis of other similar tertiary amine-based hyperbranched polymers using this strategy is also successful. When hexamethylene glycol diacrylate (HGDA) and decamethylene glycol diacrylate (DGDA) were used instead of EGDA, the Michael addition polymerization of TMEA with HGDA or DGDA reveals similar polymerization behaviors. The HypHT24 with *M*_w, triple_ = 43,300 g/mol and *M*_w_/*M*_n_ = 3.69 and HypDT24 with *M*_w, triple_ = 35,900 g/mol and *M*_w_/*M*_n_ = 2.10 are used the following studies including fluorescence investigation. Their structures were characterized; and the HypHT24 obtained by polymerization of TMEA with HGDA and the HypDT24 prepared from TMEA and DGDA have similar ^1^H NMR spectra as shown in Figure 7. The same with HypETs, both HypHT24 and HypDT24 have two terminal functional groups, acrylate and thiol, they were treated with mercaptoethanol also after the polymerization ended for improving stability during storage. Thus, we can see two proton signals of the methylene groups next to terminal hydroxyl group respectively at = 3.92 ppm (a′), which is formed by reaction of terminal SH and mercaptoethanol, and = 3.74 ppm (a), which is produced by reaction of acrylate with mercaptoethanol. Different from ^1^H NMR spectrum in Figure 5(b), Figure 7(a) shows one proton signal at = 1.65 (j) ascribed to the two methylene groups adjacent to ester methylene group, but the signal at = 1.39 ppm (k) and 1.29 ppm (k, l and m) respectively belongs to the two methylene groups and the six methylene groups in the middle of ester hexylene group for HypHT24 (Figure 7(a)) and decylene group for HypDT24 (Figure 7(b)). Similar to the HypETs, the molar ratios of HGDA/TMEA and DGDA/TMEA in the hyperbranched polymers were calculated based on the integral values of the ester methylene proton signal at = 4.09, the signals at 3.92 (a′) and 3.74 (a) as well as the signals at 2.54–3.02 ppm, the results are listed in Table 1. The molar ratios of HGDA/TMEA (1.73) and DGDA/TMEA (1.70) in the hyperbranched polymers are slight less than the corresponding value (1.80) of HypETs, which is probably related to the low concentration of reactive acrylate group in the polymerization system when the polymerization was carried out in the same monomer concentrations.

The DBs of HypHT24 and HypDT24were also calculated based on the integral values of the signals at = 3.90 (a′) and 3.74 ppm (a), and they are 0.73 and 0.70, respectively. Their DBs are also slight lower than that of HypETs, indicating higher content of linear units in the HypHT24 and HypDT24.

### Fluorescence study

As we mentioned, fluorescence of the tertiary amines has been extensively studied, but this uncommon chromophore is easily quenched [11]. Our study shows that the hyperbranched polymers with tertiary amine as branching units can retain its high fluorescence efficiency due to less mobility of the tertiary amine in the interior region of hyperbranched polymers [14], which is consistent with the result that nitrogen-incorporated in diamond can emit strong fluorescence [21]. Thus, studying the influence of ester alkylene size in diacrylates on fluorescence should be interesting because high branching density leads to a compact structure of the individual macromolecules, restricts the chain mobility, which will affect the fluorescence [22]. Three hyperbranched polymers, HypET, HypHT and HypDT respectively with ester ethylene, ester hexamethylene and ester decamethylene in diacrylate units have been synthesized as we described above. Since molecular weight of the polymers has big influence on their fluorescence [9,14], The HypET20 was selected for comparison because it has similar molecular weight with other two polymers. The fluorescence spectra in Figure 8 reveal that the fluorescence intensity has the following sequence: HypET20 > HypHT24 > HypDT24. Using Williams’ method reported [23], the quantum yields of HypET20, HypHT24 and HypDT24 were measured, and the results are shown in Figure 9. The same trend with the fluorescence intensity can be observed, probably with length increasing of the linear skeleton, the branching density decreases, leading to increase of chain mobility and decrease of the fluorescence efficiency.

## Conclusion

The tertiary amine-based hyperbranched poly(amine-ester)s have been successfully prepared via Michael addition polymerization of TMEA (B_3_) and diacrylate (A_2_), and the crosslinking reaction in the polymerization can be avoided using appropriate feed molar ratio and by control of the reaction extent. Similar to the hyperbranched polymerization of AB_2_ monomers, the molecular weight increases slowly at the initial stage of polymerization, and at the final polymerization, sharp increase of molecular weights is observed because of reactions between the high molecular weight macromolecules. The structural analysis indicates that the molar ratio of diacrylate/TMEA is lower than the feed molar ratios, DBs increase slightly with increasing molecular weights. With increase of linear spacer between the branching units, the fluorescence intensity and quantum yields decrease significantly.

## Disclosure statement

No potential conflict of interest was reported by the authors.

## Funding

This work is supported by the National Natural Science Foundation of China under [grant number 21404088]; the Fund for Outstanding Young and Middle-aged Scientists of Shandong Province under [grant number BS2014CL017]; and Shandong Provincial NSF under [grant number ZR2015EM036], [grant number ZR2015BL026].
